# Application of the Molecular Combing Technique to Starch Granules

**DOI:** 10.3390/molecules14104079

**Published:** 2009-10-12

**Authors:** Hua Li, Zhong-Dong Liu, Liu Boxiang, Jian-Hui Chen, You-Ning Sun, Xiao-Ling Lv, Ze-Sheng Zhang, Pin Sun, Pin Zhang, Yang-Li Wang

**Affiliations:** 1 Department of Food Science, Henan University of Technology, Zhengzhou 450052, Henan, China; E-Mails: lixian78101@163.com (L.H.); xiuwei1111@yahoo.cn (C.J.H.); sunyouning@yahoo.cn (S.Y.N.); zhangpin20052005@yahoo.com.cn (Z.P.); wangyanli@yahoo.com.cn (Y-L.W.); 2 Tianjin University, Weijin Road 92#, Tianjin, 300072, China; E-Mail: jollier.liu@gmail.com (B-X.L.); 3 College of Food Engineering and Biotechnology, Tianjin University of Science and Technology, No 29, the 13 Avenue of Teda, Tianjin 300457, China; E-Mails: zhangzesheng@tust.edu.cn(Z-S.Z.); sping@tust.edu.cn (P.S.)

**Keywords:** molecular combing, starch granules, nanostructure, AFM, suspected intermediates

## Abstract

The molecular combing technique was used to dissociate the nanostructural units of starch granules from the starch fragments after a gelatinization process. With the help of atomic force microscopy (AFM), we observed that some nanostructural chains were just ﬂowing out of the granules. It proves that there are substantive nanostructural units in the starch granules, a phenomenon not previously observed, so these nanostructural units were defined as suspected intermediates. Furthermore, we conclude that blocklets of starch granules are formed through twisting or distortion of nanochains.

## 1. Introduction

### 1.1. Starch granules

In the microcosm, natural starch granules are composed of 1/4- and 1/6-linked α-D-glucopyranosyl units; in the macrocosm, the diameter of starch granules falls in the micron size range [1,2]. In food science, the concept of nanostructural units composed of the polyglucopyranose chains is the core of many researches [3,4,5,6,7]. The current model conceives starch granules as the result of the gelatinization and enzymolysis of these glucopyranose chains, but there is no direct proof to confirm that substantive nanostructural units really exist in the starch granules. Furthermore, since these granular units are of nanometer size, they are a kind of nanophase materials with great potential value in diverse applications. For example, they can be used as surface-active agents owing to their large surface,. More effective practical utilization should result from a clear understanding of their structure and characteristics. 

### 1.2. The classical model of starch structure

**Figure 1:**
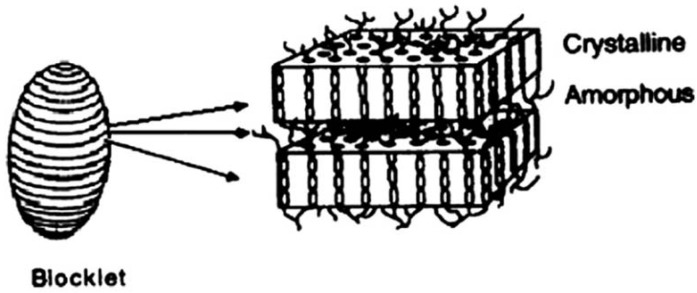
The model of blocklets.

Many data about starch structure have been obtained previously due to the continuous research for a long time. On the basis of these data Gallant *et al*. [8] put forward the classical model of starch structure in 1997. In their model, the starch granule is composed of a crystalline hard shell and a semi-crystalline soft shell. There are pores on the surface of starch granules and these pores, which are the route for soluble components to dissociate out of granules when swelling occurs and stretch into the inner parts of the granules. Both the crystalline hard shell and the semi-crystalline soft shell are made up of blocklets composed of crystalline amorphous layers (Figure 1). When large amounts of amylopectin macromolecules are assembled together, their side chains twist with each other to form a crystalline layer and their branch points (1,6-α-D-glucosidic bonds) form the amorphous layer. 

When observing the surface of starch granule with AFM, Baldwin found some protrusions [9], so in Gallant’s model [8], it was assumed that blocklets were ellipsoid in shape. In Figure 1, the egg-shaped matter on the left represents the blocklets and the rectangular block on the right hand corresponds to an amplified image of the blocklet. The middle arrowhead in Figure 1 corresponding to a line in the blocklet (left image) indicates an amorphous area in the right-hand image. The other two arrowheads (zones between the lines on the left image) point to the crystalline areas in the image on the right. The implication of this model is that a blocklet is divided into crystalline and amorphous layers and these two layers alternate with each other. However, in our experiments, we could not find any layer structures in any process of collapse and gelatinization of blocklets. Only nanochains were found, so it was deduced that a blocklet should not be composed of crystalline layer and amorphous layer, but that rather it is formed by the twisting or distortion of nanochains.

### 1.3. The technology of molecular combing

Molecular manipulation is a new technology in nanoscience. It is a kind of ﬁxed-point operation to change the uniformity of the whole macromolecule. Its main aim is to stretch the bio- and poly-macromolecules from the clew or the leptospira into straight chains in order to directly observe their structure using some special instrumentation such as AFM [10,11]. One such kind of new technology is molecular combing. In 1994, Bensimon drew out DNA chains of molecules from helixes to parallels on a microscopy glass slide with the flowing and evaporation effect of water [12]. In this study, this technique has now also been used to stretch starch nanounit chains [13].

## 2. Results and Discussion

**Figure 2:**
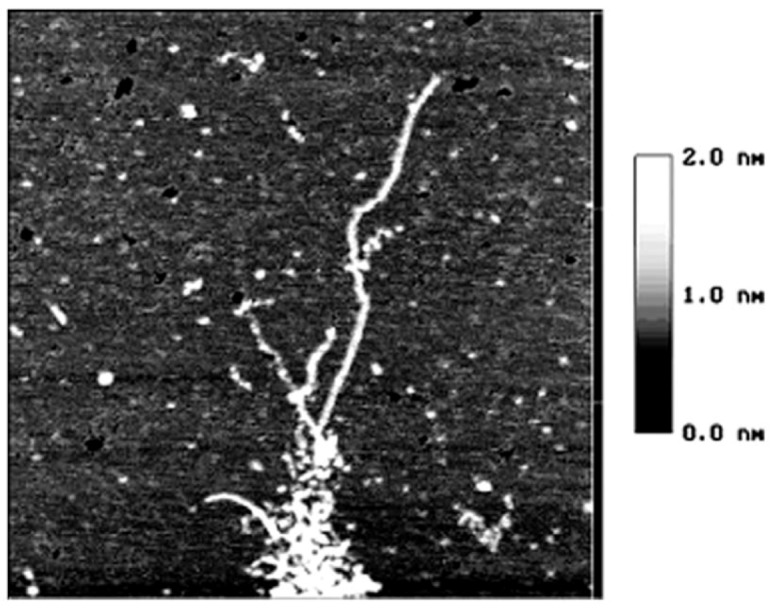
The AFM image of starch nanochains when heated in 90 °C water bath for 5 min size 1.5 × 1.5 µm [13].

**Figure 3:**
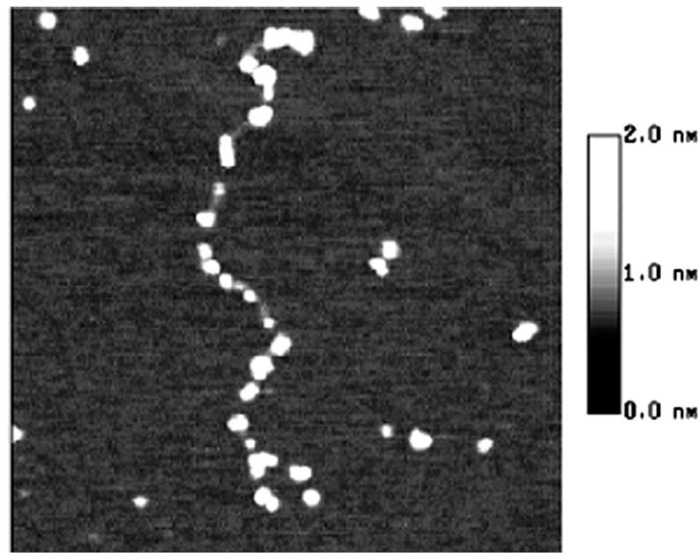
The AFM image of starch nanochains when heated in 100 °C water bath for 10 min, size 600 × 600 nm.

**Figure 4:**
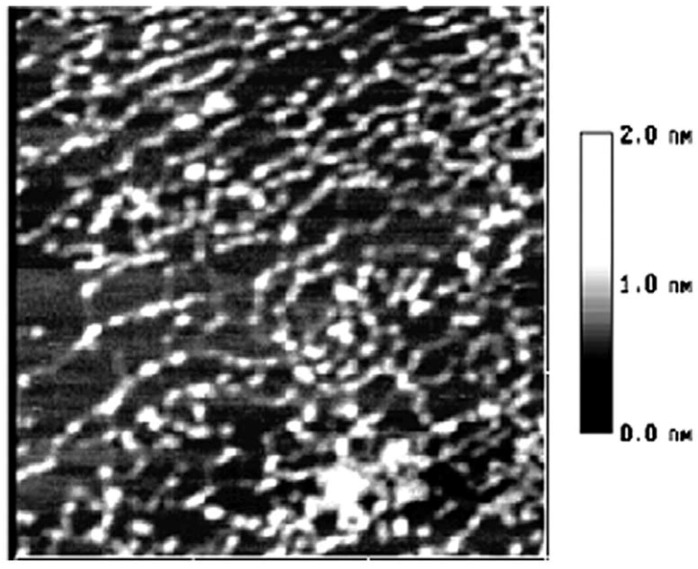
The AFM image of starch nanochains when heated in 100 °C water bath for 15 min, size 600 × 600 nm.

**Figure 5:**
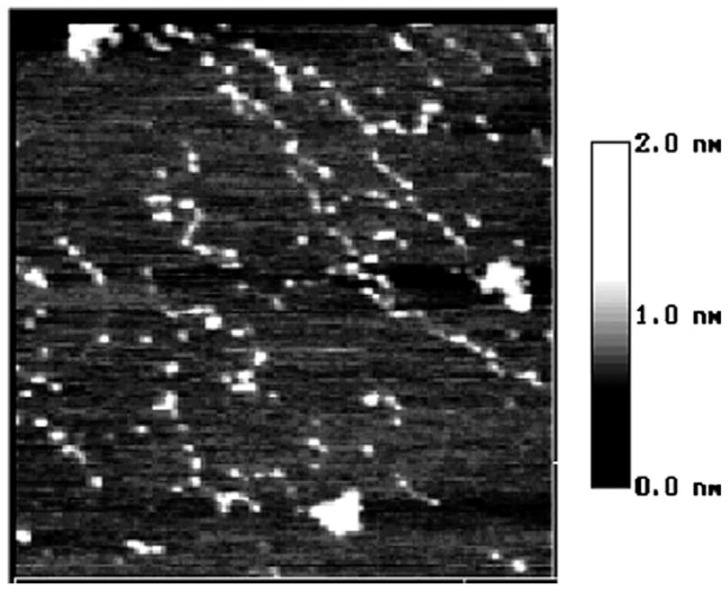
The AFM image of starch nanochains when heated in 100 °C water bath for 20 min, size 1.0 × 1.0 µm.

**Figure 6:**
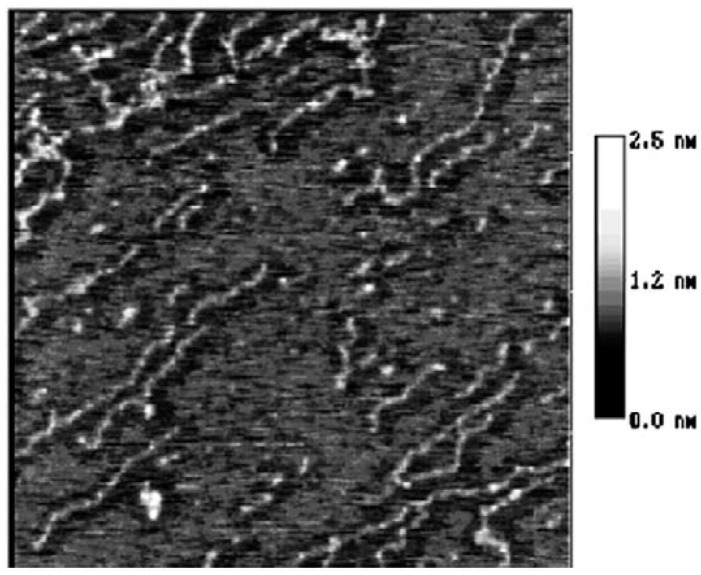
The AFM image of starch nanochains when heated in 100 °C water bath for 30 min, size 800 × 800 nm.

**Figure 7:**
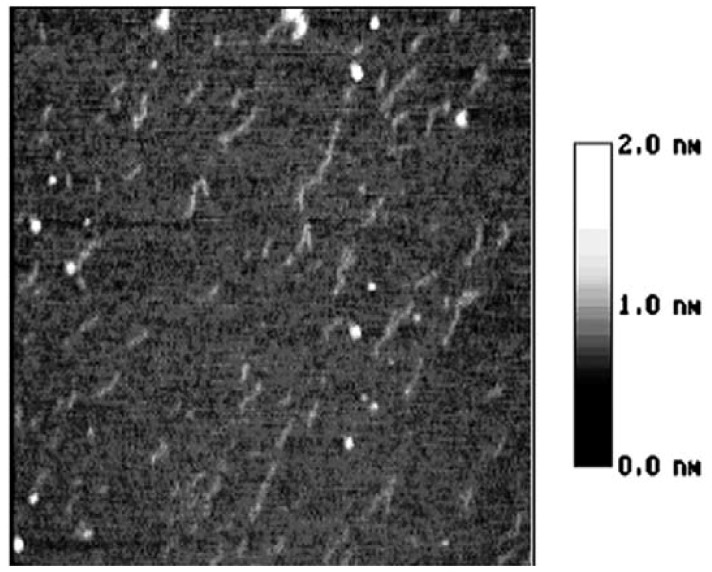
The AFM image of starch nanochains when heated in 100 °C water bath for 40 min, size 2.0 × 2.0 µm.

In this study, AFM was used to observe the chains of starch nanounits during the gelatinization process. The corresponding images can be seen in Figure 2, Figure 3, Figure 4, Figure 5, Figure 6, Figure 7, the changes in the starch nanounit chains during the gelatinization process after these chains have dissociated out of starch granules. 

Figure 2 illustrates the moment that the starch granule is just collapsing and the nanochains are just flowing out of the granules [13]. In this figure, the nanounit chains are of the same height and width and no parts of chains has reacted with water. However, when comparing Figure 3 and Figure 2, it can be observed that although the whole nanounit chain can still be identified in Figure 3, some parts of it are clearly different from others. The height and width of these parts are impaired and the whole nanounit chain is divided into salient points and conjunction areas. 

In Figure 4, the distance between salient points becomes greater and the conjunction areas are not very clear. In Figure 5, the salient points of the nanounit chains and the conjunction areas cannot be found out. In Figure 6, the nanounit chains have nearly completed the gelatinization process, so the height and width of the nanounit chains become uniform again. In Figure 7, the height of nanochains becomes much lower and the AFM image of whole nanounit chains becomes faint and blurred.

These six figures reveal the whole gelatinization process of starch nanochains. Before describing the gelatinization process from these six figures, the nanounit chains, the salient points and conjunction areas should be made clear. From the present data, the height of starch nanounit chains should be below 3.0 nm, and the diameter of blocklets should be 20–500 nm [8,16,17,18], which is larger than these nanounit chains. On the other hand, the height of starch nanounit chains (Figure 2) and salient points (Figure 3, Figure 4, Figure 5) are bigger than the helix diameter of VH-amylopectin, so these nanounit chains should not be glucose monochains. As a result, it is deduced that the starch nanounit chains in these figures should be blocklets that are stretched by the molecular combing technology. In other words, blocklets should be formed through twisting and distortion of these nanounit chains. This is different from the former classical models of starch blocklets made up of crystalline layers and amorphous layers. 

The salient points of starch nanounit chains should be the crystalline areas, which are formed by the helixes of side chains of amylopectin macromolecules. The conjunction areas of the starch nanochains should be amorphous areas, which are formed by the branch points (α-1,6-glucoside bond) of amylopectin macromolecules. This proves that starch nanounit chains contain crystalline areas and amorphous areas, alternated with each other. 

Finally we deduced the mechanism of the gelatinization process of starch nanounit chains: First the structures of the starch nanounit chains that are just flowing out of granules are very tight. The amorphous areas could not be observed by AFM and the whole nanounit chain has a uniform height and width. Next, when the nanounit chains come into contact with hot water, they can begin swelling, just like the starch granule swelling process. As a result, the structure of the nanounit chains becomes loose, and the amorphous areas can be observed with AFM. Second, gelatinization begins to happen in the amorphous areas. Water molecules penetrate slowly into the crystalline areas from the amorphous areas and untie the helixes in the crystalline locations. As a result, the crystalline areas becomes slowly disorganized and its structure becomes loose. This can explain why the height of salient points becomes lower and lower from Figure 4, Figure 5, Figure 6, Figure 7. Finally, water molecules untie the helixes of the amylopectin side chains in the crystalline areas completely and the crystalline areas vanish. The amylopectin and amylose exist in water solution as macromolecules between a few macromolecules. 

## 3. Experimental

### 3.1. Reagents and apparatus

Cornstarch (Henan Shangshui Starch Factory, Shangshui, P.R. China, ash < 0.4%, protein < 0.6%, cellulose < 0.1%); double distilled water (quartz evaporator, conductivity < 1.0 × 10^-7^ Ω·cm^-1^); Atomic Force Microscope (AFM) (Digital Instruments Co., St. Barbara, CA, USA).

### 3.2. Methods

1. Gelatinization [14]: a starch sample (0.1–0.3 mg) was placed into a covered vial (2.5 mL) and double distilled water (2 mL) was added. Then the vial was placed in a water bath 100 °C for 5, 10, 15, 20, 30 or 40 min, respectively. 

2. Molecule combing [15]: starch solution (2 mL) was deposited instantly onto the surface of newly cleaved natural mica, then the mica surface was promptly dried with a blast of air from a blastball (a normal lab tool with high elasticity that is made of rubber, so the air in it can be squeezed out as a strong airflow) in a certain single direction. It should be noted that this step must be finished within 5 s.

3. Test: all samples ready to be tested are observed using the AFM, working in tapping mode in air. 

## 4. Conclusions

In this paper the technology of molecular combing has been used to dissociate starch nanounit chains from starch fragments and these nanostructural unit chains were defined as suspected intermediates because they distributed widely in the starch fragments. From the analysis, we put forward a gelatinization model for starch nanounit chains which proves that these suspected intermediates contain crystalline areas and amorphous areas. It deduces that blocklets should be the result of the twisting or distortion of nanochains.
